# Evaluation of ultrasonic signals collected from laboratory concrete specimens: Preprocessing and analysis with coda wave interferometry

**DOI:** 10.1016/j.mex.2026.103811

**Published:** 2026-02-02

**Authors:** Eva Jägle, Rujika Tuladhar, Ernst Niederleithinger, Niklas Epple, Camila Andrea Sanchez Trujillo, Christoph Gehlen, Jithender J. Timothy

**Affiliations:** a Technical University of Munich, TUM School of Engineering and Design, Department of Materials Engineering, Chair of Materials Science and Testing, Centre for Building Materials (cbm), Munich, BY, Germany; b Bundesanstalt für Materialforschung und -prüfung (BAM), Division 8.2 NDT methods for civil engineering, Berlin, Germany

**Keywords:** Concrete technology, Ultrasound, Data evaluation, Coda wave interferometry, Microstructure

## Abstract

Efficient maintenance of infrastructure relies on monitoring and assessment of its condition. New technologies and methods thereby enable a deeper understanding of the materials used and of the structures built. Coda Wave Interferometry (CWI) is currently explored for continuous monitoring of reinforced concrete structures as well as material testing. This ultrasound-based method is sensitive to even small material alterations and therefore suitable for the detection of initial damage stages. Herein, a step-by-step procedure for the evaluation of ultrasonic signals with CWI methods is presented. The described procedure is proposed for ultrasonic signals collected with embedded ultrasonic transducers with a center frequency of 50 kHz to 70 kHz from prism-shaped concrete specimen with dimensions of 400 mm x 100 mm x 100 mm. The raw ultrasonic signal, preprocessing and CWI analysis are described and influences of parameters within the analysis are discussed. The presented procedure allows systematic and comparable analysis of ultrasonic signals generated with similar conditions and therefore contributes to the application of CWI methods for structural health monitoring and material testing.

## Highlights

• Standardized preprocessing and Coda Wave Interferometry (CWI) analysis enable low-barrier adoption for new users, ensure comparability between studies, and promote consistent terminology to avoid miscommunication.
• Detailed recommendations are provided for a specific concrete specimen size (400 mm × 100 mm × 100 mm) equipped with embedded ultrasonic transducers operating at a center frequency of around 60 kHz, as used in a long-term collaborative research project in Germany.
• The proposed workflow offers a systematic and reproducible approach to ultrasonic signal analysis, ensuring consistent results at reasonable computational cost.

## Graphical abstract

**Figure:**
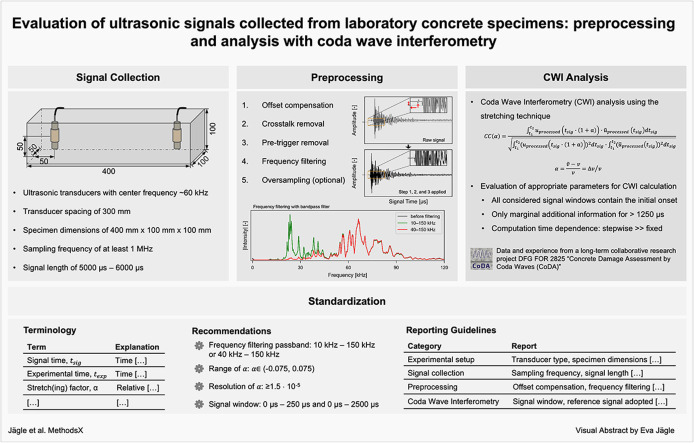

Specifications table.

**Table:** 

**Subject area**	Engineering
**More specific subject area**	Civil Engineering, Materials Science, Structural Health Monitoring
**Name of your method**	Ultrasonic Coda Wave Interferometry for Laboratory Concrete Specimens
**Name and reference of original method**	References: Snieder, R.: The Theory of Coda Wave Interferometry. Pure and Applied Geophysics 163(2–3), 455–473, (2006). https://doi.org/10.1007/s00024-005-0026-6 Sens‐Schönfelder, C., & Wegler, U.: Passive image interferometry and seasonal variations of seismic velocities at Merapi Volcano, Indonesia. Geophysical Research Letters 33(21), L21302, (2006). https://doi.org/10.1029/2006GL027797 Planès, T. & Larose, E.: A review of ultrasonic Coda Wave Interferometry in concrete. Cement and Concrete Research 53, 248–255, (2013). https://doi.org/10.1016/j.cemconres.2013.07.009 Niederleithinger, E. et al.: Processing ultrasonic data by coda wave interferometry to monitor load tests of concrete beams. Sensors, 18(6), 1971, (2018). https://doi.org/10.3390/s18061971 Fontoura Barroso, D. et al.: A Portable Low-Cost Ultrasound Measurement Device for Concrete Monitoring, Inventions, 6(2), 36, (2021). https://doi.org/10.3390/inventions6020036
**Resource availability**	The method can be implemented with any equipment and specimens that allow data to be collected and evaluated as described herein. The data used for the preparation of this manuscript are publicly available: Diewald, F. et al.: Data for: Impact of External Mechanical Loads on Coda Waves in Concrete [Data set]. Zenodo, (2025). https://doi.org/10.5281/zenodo.17880293

Alt-text: Unlabelled box dummy alt text

## Background

In recent years, the application of refined methods for the evaluation of ultrasonic signals has been explored for structural health monitoring [[1], [2]] and material testing of cementitious materials [3,4]. One approach that gained increasing attention in recent years is the so-called Coda Wave Interferometry (CWI) [5], which compares signals from a source recorded at a receiver at different points in time. From the analysis, different features can be extracted, specifically the relative velocity change Δv/v, describing the average changes of elastic properties or stiffness, and a cross-correlation coefficient *CC*, describing punctual changes in the structure causing changes in the waveform. CWI has been proven to be applicable to study even marginal material changes in concrete [6]. While fundamentals of ultrasound analysis and CWI are well documented [7], their application is part of ongoing research and standard procedures for individual applications are lacking. However, step-by-step explanations and recommendations have to be given in order to develop the methods as a tool for practical and standardized usage.

Niederleithinger et al. [8] have presented a procedure for the processing of ultrasonic signals collected from a concrete specimen with dimensions of 12 m x 0.25 m x 0.8 m. Their suggestions were followed by several studies conducted within a long-term collaborative research project in Germany [9], where experiments at the specimen scale (height + width + length < 1 m) [10,11] and structural scale (height + width + length > 1 m) [[12], [13], [14], [15], [16], [17]] were carried out.

Following this procedure, the raw ultrasonic signals are first preprocessed by applying offset compensation, crosstalk and pre-trigger removal, and band pass filtering. Subsequently, CWI methods are applied to the preprocessed signals. While existing studies contain individual investigations, details of the analysis procedure have not been developed into a standard operation procedure which is required for widespread application in materials testing. This applies not only to preprocessing specifications such as the use of band pass filters, but also to the boundary conditions that must be set when deriving the relative velocity change ∆*v*/*v* which maximizes signal correlation during CWI analysis. The values obtained by CWI analysis are sensitive in particular to the range and resolution of *α* considered when maximizing the correlation coefficient max(CC(α)), with α=Δv/v, and the signal time window $t_{sig,1}$ to $t_{sig,2}$ used.

Here, a procedure for preprocessing ultrasonic signals and subsequent application of CWI methods collected from specimen scale concrete samples with the dimensions of 400 mm x 100 mm x 100 mm is presented. Thereby, for individual steps of the analysis procedure recommendations are given regarding the parameters which need to be determined for calculation. The information is provided as a guide for scientists and engineers working at universities and engineering offices who are experts or newcomers to the field of ultrasonic testing with Coda Wave Interferometry. While the recommendations mainly refer to the application of the method on a smaller laboratory scale, they are also intended to serve as a first reference point for application and standardization in structural monitoring and are therefore also of relevance to infrastructure owners, and technical and standardization committees.

## Method details

**Fig. 1:**
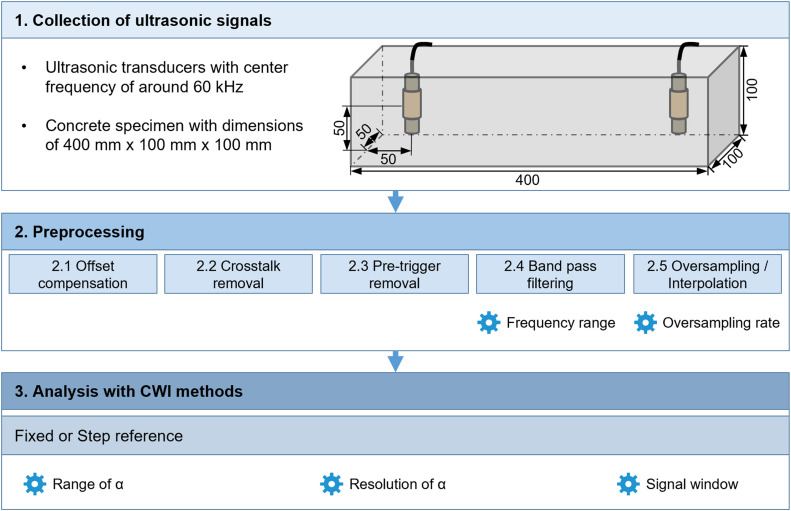
Flow diagram of the proposed evaluation method. For each step, parameters which need to be determined for calculation are indicated by a gear symbol.

The method follows the procedure proposed by Niederleithinger et al. [8] and is refined for the specified experimental setup. The workflow consists of 1. Collection of the raw ultrasonic signals 2. Preprocessing of the raw signals and 3. Application of CWI methods to preprocessed ultrasonic signals. A schematic overview is given on Fig. 1.

## Terminology

The following terms are adopted for the description of the method: Table 1

**Table 1:** Definitions of technical terms.

Term	Explanation
Ultrasonic signal, $u(t_{sig})$	Time series of ultrasonic wave amplitudes as a function of signal time (mostly: acceleration, sometimes velocity) (NDT: A-scan, geophysics: trace). Note: the term signal time (*t_sig_*) is used here for the time axis of a specific signal (duration: some milliseconds), where *t_sig_* = 0 refers to the emission of the source signal. There is a second time axis experimental time (*t_exp_*) which describes the time over a monitoring experiment (mostly hours to years) with many signals collected.
Signal window, $u{\left(t_{sig}\right)}_{t1}^{t2}$	Part of the signal used for CWI evaluation, expressed as a time window from *t*1 to *t*2.
Raw ultrasonic signal, $u_{raw}\left(t_{sig}\right)$	Time series of ultrasonic signal as recorded by the measurement device.
Preprocessed ultrasonic signal, $u_{preprocessed}\left(t_{sig}\right)$	Time series of an ultrasonic signal after all preprocessing steps were applied.
Signal time, *t_sig_*	Time within the time series of a single ultrasonic signal. Represents the time axis of a recoded waveform.
Experimental time, *t_exp_*	Time at which an individual ultrasonic signal is collected during the experiment/monitoring series. Value can be given in date/time format or absolute times.
Samples, *n*	Data points in a single ultrasonic signal. The number of samples and the sampling frequency together determine the duration of the recorded signal.
Stretch(ing) factor, *α*	Relative temporal signal dilation or compression applied during CWI analysis. Note: Some literature uses ε, which should not be confused with material strain ε.
Range of $\alpha ,\alpha \in [\alpha_{min},\alpha_{max}]$	Range of stretching factors evaluated in the CWI analysis.
Increments of *α*	Number of discrete steps considered within the range of *α*.
Resolution of *α*	Resolution considered in the CWI for the Range of *α*: Range of *α* / Increments of *α*.
Maximized Correlation Coefficient *CC_max_*	Highest correlation value max(CC(α)) obtained between a dilated or compressed version of the reference signal and another signal collected at a later experimental time. Indicator for inhomogeneous, often irreversible changes.
Relative velocity change, Δv/v	Equivalent to the signal dilation or compression *α* that maximizes the cross correlation between two signals ($CC_{max}$). Indicator for homogeneous, often reversible changes such as a moderate change in stress or temperature. Notation should use the upper-case delta.
Remaining Decorrelation $DC_{r}$	Remaining decorrelation for optimal stretching ($DC_{r}=1-CC_{max}$). Indicator for inhomogeneity and additional scatterers, e.g. cracks.
Fixed reference	Indication of how signals are compared during CWI. Here, all signals are compared to the same reference signal recorded at the beginning of the observation period.
Step(wise) reference	Indication of how signals are compared during CWI. Here, adjacent signals are compared such that the previous measured signal is taken as the reference for comparison. Cumulation of Δv/v is necessary to retrieve overall change since the beginning of observation.
Moving reference	Indication of how signals are compared during CWI. Here, fixed and step reference are combined, such that the reference signal is only updated, once $CC_{max}$ drops below a certain threshold. Cumulation of Δv/v is necessary to retrieve overall change since beginning of observation.

## Collection of ultrasonic signals

The presented analysis procedure is specified for the evaluation of ultrasonic signals collected with symmetrically embedded cylindrical piezo-ceramic ultrasonic transducers with a center frequency of 50 kHz – 70 kHz [18] from concrete specimen with dimensions of 400 mm x 100 mm x 100 mm. The transducers are embedded 300 mm apart. This setup has been used in previous studies [[10], [11]] and was developed as part of fundamental research for the application of CWI methods to reinforced concrete structures [8]. With a concrete specific ultrasonic pulse velocity $v_{p}$ around 4300 m/s and respective ultrasonic shear wave velocity $v_{s}$ of about 2500 m/s [7] wavelengths of around 61 mm to 86 mm (p-waves) are achieved and the distance of the sender and receiver equals three to five times the wavelength. It should be noted, that the specific frequency spectrum varies slightly from transducer to transducer and center frequencies stated in technical data sheets usually refer to a reference-medium, e.g., water as used in [18], and embedding in concrete generally leads to slightly higher frequencies. While these minor variations do not affect the signal evaluation presented here, every publication should include a frequency spectrum of the raw signal collected.

The sensors used for this study have been specifically developed for embedding in concrete for permanent monitoring and have been applied in related studies for years. They are the only transducers available commercially. The main focus in the development was a good balance between signal strength and frequency, as well as the ease of installation and permanence of constant coupling.

Different experimental geometries or limited sensor availability can require the use of different sensor types. In this case it is recommended to check the frequency range [19]. In case of external coupling, the stability of the coupling method, e.g., thermal stability of the coupling agent or stability of mechanical coupling method, for the projected experimental period has to be validated. For different specimen geometries, a sensitivity analysis and or simulation will be beneficial.

In this study, the following procedures have been developed for data collected by a measurement device such as the one described by Barroso et al. [20] or similar, with a sampling frequency of at least 1 MHz. To achieve a high signal-to-noise ratio, the measurement device can amplify the source signal sent to the transmitter to up to 300 Vpp (Volt peak-to-peak). This also enables monitoring on different scales with greater distances between the source and receiver than the 300 mm used here.

As it is feasible to use only a part of the signal (signal window) for analysis (see Section 3.2.), it is recommended to record signals with a length of 5000 µs to 6000 µs. In larger objects, longer recording times may be required. It is recommended to set the length to a time when the ultrasonic amplitudes are attenuated below noise level. It is also recommended to record 50 – 200 samples before source trigger onset $t_{sig}=0$ (so-called pre-trigger samples), which allow to assess the noise level, offset and drift. For a sampling frequency of 1 MHz this corresponds to 100 µs – 200 µs.

To improve the signal-to-noise ratio, some measurement systems acquire multiple signals during a single measurement event and combine them into a single representative signal, for example by averaging the individual recordings. How many signals are averaged can typically be adjusted by the user. As such signal processing performed by the measurement system can influence the quality of the obtained data, all details and settings should be reported. In the presented case, the raw signals correspond to an average of 10 individual recordings.

The presented approach can be applied to specimen setups with differing specimen shape, sensor types, and signal collection parameters with various measurement tools. In these cases, however, the procedure should be validated again before application.

The ultrasonic signals, which are analyzed using the procedure presented here are generated within laboratory experiments. During the experiments, signals are collected before, during, and after the application of loads (e.g., mechanical load, temperature, humidity) on the specimen. Signals are therefore recorded repeatedly from the same specimen and same transmitter–receiver combination creating a time series of time series, i.e., a collection of ultrasonic time-domain waveforms collected at different times during the monitoring period. The experimental time $t_{exp}$ refers to time points within a single experiment when a specific signal is recorded whereas the time $t_{sig}$ refers to the time series of one signal.

## Preprocessing

Before application of CWI, the ultrasonic time series signal requires preprocessing. Preprocessing is applied and compensated for instrumentation effects and for noise reduction. Preprocessing follows the workflow presented by Niederleithinger et al. [8] including offset compensation, crosstalk removal, pre-trigger removal, band pass filtering and oversampling/interpolation. In the following sections, each step is described in detail and the changes made in the signal are presented.

Additional preprocessing may be necessary if the signal quality or features differ from those shown here. One useful step is tapering, where a function is applied which gradually decreases to zero at the edges of the time signal. This helps reduce discontinuities between the start and end of the signal, which is an important consideration before filtering, especially when the signal lacks smooth edges, e.g., when crosstalk overlaps with the beginning of the signal or when the signal’s start and end values differ. A best practice for tapering and further reading can be found summarized in [21].

### Raw signal

**Fig. 2:**
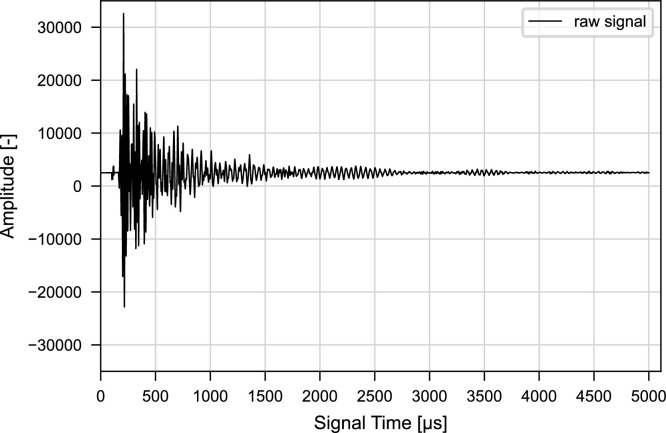
Raw ultrasonic signal before processing. Signal collected with a sampling frequency of 2 MHz taken from [[10], [22]].

The raw ultrasonic signal represents the signal as recorded from the measurement device. The time series shows the amplitude of the ultrasonic signal over signal time as illustrated in Fig. 2.

### Offset compensation

First, the amplitude offset of the signal is compensated. This is achieved by subtracting a constant correction value to each point of the signal, such that the signal is oscillating around zero. Therefore, the mean value for one predefined signal window of the raw signal $u_{raw}{\left(t\right)}_{t1}^{t2}$ is calculated and subtracted from the whole raw signal $u_{raw}\left(t\right)$ according to Eq. (1). The resulting offset compensated signal $u_{off}\left(t_{sig}\right)$ is retrieved using the mean value (alternative: median value) of the pre-trigger samples with the lower and upper boundary $t_{1}^{off}$ and $t_{2}^{off}$. For the measurement setup employed here pre-trigger length of 100 µs are used corresponding to $t_{1}^{off}=0$ and $t_{2}^{off}=100$.

(1) $$u_{off}\left(t_{sig}\right)=u_{raw}\left(t_{sig}\right)-mean\left(u_{raw}{\left(t_{sig}\right)}_{t_{1}^{off}}^{t_{2}^{off}}\right)$$

Certain measurement setups, while not the ones discussed here, also show linear drift, i.e., change of offset over signal time. This can be compensated by fitting a line of the form $u=a\cdot t_{sig}-b$ to the first and the last samples of the signal and calculating correction values for each signal according to Eq. (1). Note, that offset and drift compensation can also be performed by a high pass or band pass filter (Section 2.4). However, using the steps reported here it is recommended to track signal quality.

Further, some systems for signal collection perform offset compensation before saving the signal. For such systems, the offset should also be examined to ensure that compensation has been performed correctly and, if in question, carried out again.

### Crosstalk removal

When the signal is triggered at the transmitter, electromagnetic crosstalk is often recorded at the receiver due to the high voltage of the transmitted signal. The crosstalk (if present) is visible in the initial part of the signal and should be removed to prevent bias in the CWI analysis. Therefore, the values of the signal within the signal window representing the crosstalk are set to zero as described in Eq. (2). Here, the lower and upper boundaries of the signal window of the crosstalk $t_{1}^{ct}$ and $t_{2}^{ct}$ need to be chosen appropriately by inspection of the raw signal.

For the presented cases the crosstalk occurred within the first 125 µs of the recorded signal. Therefore, the signal without crosstalk $u_{off\_ct}\left(t_{sig}\right)$ is calculated by using $t_{1}^{ct}=0\mu s$ and $t_{2}^{ct}=125\mu s$.

(2) $$u_{off\_ct}\left(t_{sig}\right)=0fort_{1}^{ct}\leq t_{sig}\leq t_{2}^{ct}$$

Note that on certain occasions, especially for short source receiver distances, the crosstalk can overlap with the actual ultrasonic signal. The crosstalk window therefore must be set for each pair of transducers and can differ from each other. In cases where an overlap is observed, it is recommended to taper the signal after the zeroed section to generate a smooth onset of the signal. However, it is mandatory not to use the affected part of the signal for CWI analysis. As discussed above, this does not occur for the cases presented here and tapering is therefore not included as an additional preprocessing step.

### Pre-trigger removal

After offset compensation and crosstalk removal, the pre-trigger, i.e., the signal window which was recorded prior to triggering the ultrasonic pulse, is removed. The result of this part of the processing is a signal that is shorter than the raw signal. For this, the lower and upper boundary of the removed signal window $t_{1}^{pt}$ and $t_{2}^{pt}$ are chosen such that $t_{1}^{pt}=0\mu s$ and $t_{2}^{pt}$ covers the pre-trigger range.

As described above, a pre-trigger length of 50 µs to 100 µs (50 samples at 1 MHz and 200 samples at 2 MHz) is recorded for the cases described here. Therefore, $t_{1}^{pt}=0\mu s$ and $t_{2}^{pt}=100\mu s$ is used to obtain the shortened signal $u_{off\_ct\_pt}\left(t_{sig}\right)$ according to Eq. (3).

(3) $$u_{off\_ct\_pt}\left(t_{sig}\right)=u_{off\_ct}{\left(t_{sig}\right)}_{t_{1}^{pt}}^{t_{2}^{pt}}$$

**Fig. 3:**
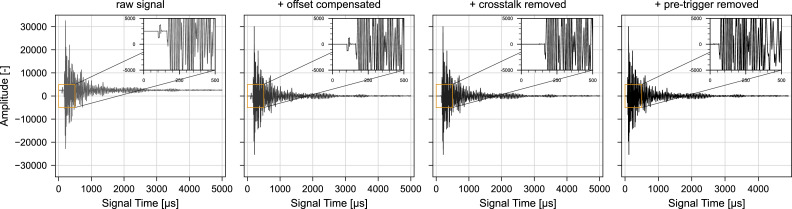
Ultrasonic signal collected with embedded 60 kHz transducers inside a concrete specimen with dimensions of 400 mm x 100 mm x 100 mm subjected to compression. From left to right: Raw signal, signal after offset compensation, signal after offset compensation and crosstalk removal, and offset compensated signal with removed crosstalk and pre-trigger. Raw signal from [[10], [22]].

In Fig. 3, the changes made in the ultrasonic signal during offset compensation, crosstalk correction and pre-trigger removal are illustrated for a signal collected by Diewald et al. [[10], [22]].

### Bandpass filtering

A zero-phase bandpass filter is used to remove frequencies outside the targeted range, e.g. to suppress electronic noise. At least the frequencies outside the sensitivity range of the transducers (here: 20 kHz to 100 kHz) should be removed. The bandpass filter is a function which passes frequencies between the lower bandpass frequency $f_{low}$ and the upper bandpass frequency $f_{up}$ and suppresses the frequencies which are outside the defined range. The Butterworth Band Pass filter is designed to have a flat frequency response within the passband, to preserve the ultrasonic signal characteristics but to exclude noise with a different frequency content. Butterworth Band Pass filters have a smooth roll-off, therefore, they show no ripple effects on the passband nor the stopband. When implemented, the filtering must ensure no phase shifts, therefore it is necessary to apply the linear filter twice, once forward and once backwards to achieve the zero phase. This can be implemented for example with the filtfilt function from the SciPy package in Python [23]. An example for the benefit of frequency filtering is given in [24]. Changes are made in the frequency domain, and after a subsequent inverse Fourier transform, the preprocessed signal $u_{preprocessed}\left(t_{sig}\right)$ is obtained as a time series.

Further, it should be noted that different frequencies are sensitive to different types of material changes. In concrete, higher frequencies are more sensitive to spatially smaller perturbations but attenuate fast, low frequencies are sensitive to spatially bigger perturbations only, but measurements can be made with larger transmitter-receiver distances. By removing certain frequencies, others are given more emphasis. Filtering, therefore, is a powerful tool to vary the type of extracted information. For this reason, not only the frequency spectrum of the raw signal but also the frequency spectrum of the filtered signal should always be reported.

**Fig. 4:**
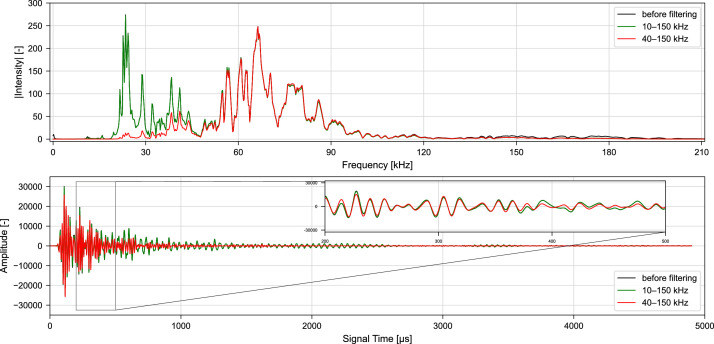
Frequency spectrum (top) and time series (bottom) of an ultrasonic signal before and after bandpass filtering. Before filtering the preprocessing steps as described in Sections 2.1.–2.3. were applied. Data from Diewald et al. [[10], [22]].

To achieve comparability between studies and to provide a basis for initial evaluations, two frequency ranges are proposed for the experimental setup described here. In Fig. 4 the time series and frequency spectrum of an ultrasonic signal before and after filtering is illustrated for both suggested frequency bands.

First, a bandpass with a frequency range of 10 kHz – 150 kHz can be applied, rejecting frequencies outside the sensitivity range of the transducers but without any further alteration within. This is in line with Niederleithinger et al. [8], which have used exactly that range. Since the setup described here is subject to very specific, reproducible conditions (sample size and geometry, transducer type, etc.), it is assumed that comparable frequency spectra are recorded always. Minimizing signal modification therefore provides comparability without filter-induced bias.

Alternatively, a bandpass with a frequency range of 40 kHz – 150 kHz can be applied, rejecting the frequencies around 25 kHz which are observed with significant energy for the specimen setup described here. As described in Section 1, the transducers are of cylindrical shape with a piezo-ceramic element, emitting ultrasonic pulses mainly orthogonal to the main axis of the cylinder at a center frequency of 50 kHz – 70 kHz. However, during pulsing, the transducer also deforms along the main axis with frequencies around 25 kHz. It has been observed that these frequencies are recorded with significant energies from smaller concrete specimens (400 mm x 100 mm x 100 mm) but not in larger specimens, and this effect is therefore attributed to the small distance between the transducers and the specimen surfaces. When using different transducers with a similar center frequency around 50 kHz –70 kHz, the frequencies around 25 kHz will not be recorded with the same intensity. Therefore, it is suggested to remove these frequencies for comparability with studies relying on other types of sensors.

### Oversampling

Preprocessing may be completed by oversampling the ultrasonic signal. Oversampling can be achieved in the frequency domain by zero-padding the signal’s spectrum and transforming back to the time domain. This approach yields smooth and accurate intermediate samples while preserving the original spectral content. Oversampling of signals recorded with 1 MHz to 10 MHz can improve the CWI analysis, especially when very small increments of *α* are considered. However, oversampling is not always required for a sufficient quality of the results and therefore could be omitted during analysis for shorter processing times based on tests done on selected signals.

## Analysis with coda wave interferometry

Coda Wave Interferometry (CWI) relies on the comparison of a reference signal, recorded at an initial material state and a perturbed signal recorded after the material has undergone changes. The coefficient of cross-correlation (*CC*) or similar properties such as coherency between the ultrasonic signals can be computed to assess the changes in the material qualitatively. Several approaches have been proposed for quantifying the signal changes.

Here, the stretching technique [[8], [25]] is applied, where the reference signal $u(t_{sig})$ is stretched or compressed linearly and for each stretching factor *α*, the correlation coefficient CC(α) between the stretched reference signal and another signal of interest $\tilde{u}\left(t_{sig}\right)$ is calculated according to Eq. (4). In many cases, just a part of the signal between *t*_1_ and *t*_2_ (time window $u{\left(t_{sig}\right)}_{t1}^{t2}$) is used to exclude direct (ballistic) waves, to exclude parts of the signal dominated by noise, or to control spatial sensitivity (early part: area close to direct path, later parts: scattered, longer paths):

(4) $$CC\left(\alpha \right)=\frac{\int_{t_{1}}^{t_{2}}u_{preprocessed}\left(t_{sig}\cdot \left(1+\alpha \right)\right)\cdot {\tilde{u}}_{preprocessed}\left(t_{sig}\right)dt_{sig}}{\sqrt{\int_{t_{1}}^{t_{2}}{\left(u_{preprocessed}\left(t_{sig}\cdot \left(1+\alpha \right)\right)\right)}^{2}dt_{sig}\cdot \int_{t_{1}}^{t_{2}}{\left({\tilde{u}}_{preprocessed}\left(t_{sig}\right)\right)}^{2}dt_{sig}}}$$

During computation, the stretching factor *α* is varied in small increments such that CC(α) is maximized. The stretching factor which yields the highest correlation between the reference and perturbed state signal, i.e., $CC_{max}$, is related to the relative velocity change Δv/v between the velocity of the reference signal *v* and the velocity of the other signal $\tilde{v}$ according to Eq. (5):

(5) $$\alpha =\frac{\tilde{v}-v}{v}=\Delta v/v$$

Negative Δv/v values indicate a velocity decrease in the changed state compared to the reference state, while positive Δv/v values indicate increase in velocity. Note, that there are different notations in literature where (1−α) is implemented in Eq. (4), such that α=−Δv/v. The derivation of (4), (5) is provided in the appendix.

In literature, the notations for the relative velocity change further vary with regards to whether lower- or upper-case delta (∆ or *δ*) or simply a lower-case Latin d is used and whether the letter v is written italic or upright. In line with standardization, e.g. [26], the notation Δv/v with capital delta and italic v should be adopted as Δ indicates a finite difference, whereas δ or d denote small or infinitesimal variation and italic letters are used for symbols representing physical quantities while upright letters are used for units.

The velocity change Δv/v is usually used as an indicator for a homogeneous, often almost completely reversible change such as a moderate change in stress or temperature. The remaining decorrelation $DC_{r}$, with $DC_{r}=1-CC_{max}$, is an indicator a) for inhomogeneity and b) for additional scatterers, e.g. cracks.

**Fig. 5:**
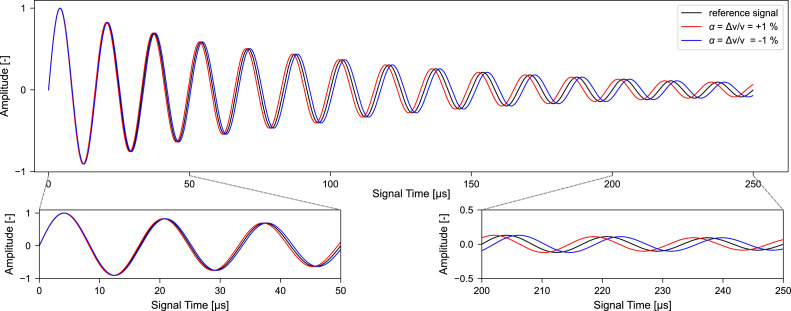
Idealized wave form with no stretching (reference signal) and stretching by ±1 % applied. A stretching factor of *α* = +1 % indicates that the reference wave needs to be compressed to match the changed wave form (velocity increase). Analogously, a stretching factor of *α* = −1 % indicates that the reference wave needs to be dilated to match the changed wave form (velocity decrease).

Signal alterations due to dilation and compression by 1 % are shown in Fig. 5 using an idealized waveform for illustration purposes. The initial reference signal is synthetically generated as a damped sinusoidal waveform incorporating smooth amplitude modulation. The idealized signal was generated with a 10 MHz sampling rate over a duration of 2048 μs, using a 60 kHz frequency. It incorporates an exponential decay with a rate of −10,000 μs⁻¹, a modulation depth of 0.3, and a smoothness parameter of 100 to produce realistic amplitude modulation.

### Fixed, step(wise) and moving reference

When analyzing a set of signals recorded, e.g., during an experiment, three approaches can be used to derive the signal changes over time:

(1) *Fixed reference*: All signals are compared to the same reference signal recorded at the beginning of the observation period. For each signal, the relative velocity change Δv/v reflecting the change between the initial state and the state at signal collection is retrieved. A fixed reference is suggested when the changes in the medium are small and $CC_{max}$ value stays above 0.7. This value is derived from observation and experimentation; other authors recommend even stricter limits.
(2) *Step(wise) reference*: Calculations are performed for adjacent signals such that the previous measured signal is taken as the reference for comparison. Therefore, the stretching yields the relative velocity change Δv/v between two adjacent signals. For derivation of the signal change with respect to the initial state, the velocity variation needs to be cumulated. The cumulated stretching factor $\alpha_{c}$ or cumulated relative velocity change Δv/v, for the n^th^ signal can be calculated as given in Eq. (6) and is derived as described in the appendix. This approach has been used in various previous studies, e.g., by Niederleithinger et al. [8] and Sun et al. [27]. Using the stepwise reference approach is suggested, when signals exhibit major changes with $CC_{max}$ of the fixed reference approach falling below 0.7.
  (6) $$\alpha_{c,n}=\{\begin{array}{c} \alpha_{n},\\ \left(\alpha_{c,n-1}+1\right)\left(\alpha_{n}+1\right)-1, \end{array}\begin{array}{c} n=1\\ n>1 \end{array}$$
  Attention must be paid when applying Eq. (6), as rounding errors may occur, which also accumulate and may lead to significant deviations between the actual and calculated values. The relative deviation between actual and cumulated value is potentially greater if the change per adjacent signal is small and many individual values are cumulated. This is why, in the stepwise method the choice of an adequate increment of *α* (see Section 3.2) is crucial, as bigger step size leads to inaccuracies in the calculation of the velocity change, and error propagation cumulates to large deviations from the actual velocity change. To test whether accumulation of rounding errors occurs in a certain analysis, additional calculations may be performed and compared for stability of the results, e.g., using a subset of the collected signals and fixed reference approach, or by skipping signals when computation with the stepwise reference approach. Note, that CC values can’t be accumulated.
(3) *Moving reference* (or auto CWI): Fixed and stepwise reference can be combined, such that the reference signal is only updated, once $CC_{max}$ drops below a certain threshold, e.g., 0.7. This approach was applied, e.g., by Diewald et al. [10] and Vu et al. [28] who studied mechanical loading of concrete specimen and observed periods of small signal changes where fixed reference can be applied and periods of strong signal alterations, where consecutive signals needed to be compared to maintain sufficient correlation.

Using moving reference cumulation of the relative velocity change according to Eq. (6) needs to be performed to relate the values back to the initial state each time the reference is updated. However, as mentioned above, CC values cannot be accumulated, implying that the resulting values for $CC_{\text{max}}$ only refer to the state of the last update. The number and distribution of updates over the duration of the monitoring thereby depends on the selected threshold. Therefore, the results retrieved using moving reference should always include an indication when updates were made.

As discussed above, both values, Δv/v and $CC_{\text{max}}$, provide different valuable information. While both parameters for the fixed and stepwise approach can be interpreted consistently over the entire monitoring period, interpretation of the moving reference results is more challenging and may lead to misinterpretations, especially by inexperienced users. Therefore, it is recommended to apply either fixed reference or stepwise reference to all analyzed signals consistently, since this allows clear interpretation of the retrieved parameters. Both approaches should be applied complementary for analysis of the entire monitoring period or for specific subperiods of interest to assess the true characteristics of signal changes.

### Analysis parameters

For implementation of the analysis and calculation of the CWI parameters, Δv/v, or cumulated Δv/v and $CC_{max}\left(\alpha \right)$, the following parameters need to be selected: (1) signal window $u{\left(t_{sig}\right)}_{t1}^{t2}$, (2) range of *α*, and (3) resolution of *α*. A description of the individual parameters and their influence on the values obtained is provided below.

#### Signal window

The maximum signal window length is determined by the measurement settings. As stated in Section 1, a raw signal length of 5000 µs – 6000 µs is recommended for our sample size. After preprocessing, the signals are shortened by 100 µs (see Section 2), with new signal time $t_{\mathrm{sig}}=0\;\mu s$ corresponding to the signal triggering at the transmitter. Any signal window from 0 µs ≤ *t*_1_ < *t*_2_ ≤ signal length can be used for calculation according to Eq. (4).

Whether the full signal or distinct windows should be implemented depends on the purpose of the analysis. The signal window may or may not cover the initial onset of the signal (direct wave). When including the direct wave, it must be taken into account that this signal part has greater emphasis on the calculated values compared to later signal times with lower signal amplitude. The choice of a suitable time window is specific to the experiment geometry, measurement setup and the material changes studied. It is recommended to perform a sensitivity analysis with a diffusive [29], radiative transfer [30], or simulation based approach [31] for new experimental studies, to decide on the analysis window.

Note, that it is a matter of debate whether the analysis can be referred to as Coda Wave Interferometry, if beside the late part of the signal (“coda”), the initial ballistic (direct) part is also included. If the entire waveform or the initial part together with values recorded after the main peak are used, other terms such as (full) wave comparison might be adopted. Here, the term Coda Wave Interferometry is assumed to be applicable, provided that Eq. (4) and signal windows including a coda component, i.e., a signal part containing information from scattered waves, is used. This definition is particularly useful as there is no common definition of where the coda begins.

To ensure comparability of ongoing and future research efforts, the signal windows of 0 µs – 250 µs, 0 µs – 1250 µs, and 0 µs – 5000 µs are suggested for analysis of signals collected with the setup specified in Section 1. For efficient initial analysis it is recommended to be performed with fixed reference using a signal window of 0 µs – 250 µs, 0 µs – 1250 µs and 0 µs – 5000 µs and stepwise reference analysis with a signal window of 0 µs – 250 µs and 0 µs – 1250 µs. Within the 0 µs – 250 µs period, the p-waves will travel the specimen 2 – 3 times and the s-waves will travel the length 1 – 2 times with the signal window covering the maximum amplitude peak but cutting off shortly thereafter. The broader window of 0 µs – 1250 µs period covers enough time for the p-waves and s-waves to travel the length around 13 times and 8 times, taking into account the main peak and later arrivals, until the signal amplitude falls >80 % below the main peak. A discussion of the meaning of the window with respect to data interpretation when using the sample setup as presented here can also be found in Vu et al. [28].

#### Range of *α*

Finding a value for *α* that maximizes *CC*(*α*) (corresponds to finding *CC_max_*, the highest correlation between the stretched signal and the reference) proposes a parameter estimation problem for which the solution space must be pre-defined to ensure that a solution is found. The range of possible values for *α* must be chosen such that it includes the true value to be found. If the true relative velocity change between two signals is outside of the defined range of *α*, calculations will provide a solution which does not reflect the true value.

The suggested range of *α* implemented for analysis depends on the expected relative velocity variation. Previous studies indicate velocity changes around 0.1 %/MPa for compressive loads and 0.3 %/MPa for tensile loads in the elastic range [10], as well as around 0.4 %/K and 0.03 %/ %RH for changes in ambient temperature and relative humidity [[10], [11]]. Both studies relied on the same measurement set up as described herein. The relative velocity change during mechanical compressive and tensile loading until failure, as well as for temperature variations of 5 °C – 55 °C and relative humidity changes of 35 % – 95 % were within ±6.0 %. A range of *α* with α∈(−0.075,0.075) is therefore suggested for initial analysis of signals.

#### Resolution of *α*

In addition to setting of the lower and upper boundary values of the range of *α*, indications must be given for the values to be considered within this range. This can be implemented by defining the number of steps to be evaluated when screening the range of *α* for finding $CC_{max}$. The true relative change in velocity can only be found using the CWI if the increments contain at least approximately the true value. The resolution of *α* as the range of *α* divided by the number of considered steps minus 1 therefore influences the accuracy of the results.

Although finer resolution increases the likelihood of finding the true value, it does also increase computational costs (see also Fig. 12). For relative velocity changes detectable by CWI a resolution on 2∙10^–5^ was indicated by Larose and Hall [32]. For the presented application case with a range of *α* with α∈(−0.075,0.075) a resolution of *α* with 10,001 increments is suggested, resulting in a resolution of 1.5∙10^–5^. For some cases the use of even finer resolutions of up to 1∙10^–6^ might be advantageous [33]. It is also possible to start with a coarse resolution and to refine later; but this approach should be validated carefully and is not used here.

## Data comparison for interpretation

The obtained relative velocity change Δv/v and maximized correlation coefficient $CC_{max}$ are functions of the material changes occurring in the monitored specimen or structure. Deriving the material change of interest, e.g., crack formation, requires separation from other changes, e.g., thermal strain. This can be achieved by comparison of the Δv/v and $CC_{max}$ values with supplementary information, e.g., temperature recordings. The derivation of general relationships between exposure, material change, and CWI parameters is subject of ongoing research.

## Reporting

Reporting signal collection, preprocessing and CWI analysis for scientific publications should include the following information: Table 2

**Table 2:** Reporting signal collection, preprocessing and CWI analysis for scientific publications.

Data collection and analysis properties and settings			Example
Experimental setup	**Transducer type and specifications**	Report the sensor type, center frequency, and coupling conditions.	Symmetrically embedded cylindrical piezo-ceramic ultrasonic transducers with a center frequency of 60 kHz.
	**Specimen dimensions and geometry**	Specify the size and shape of the specimen(s).	Prism-shaped concrete specimens with dimensions of 400 mm × 100 mm × 100 mm.
	**Transducer spacing**	Report the distance between transmitter and receiver.	Transducers were embedded 300 mm apart.
	**Measurement Device**	Describe the data acquisition system.	A measurement device similar to the one described by Barroso et al. [20] was used.
Signal collection	**Measurement frequency**	Report the interval between individual measurements.	Signals were collected every 10 min.
	**Sampling frequency**	Report the frequency used for signal sampling.	A sampling frequency of 2 MHz was used.
	**Signal recording duration**	Specify total signal length.	Signals were recorded with a length of 5000 µs.
	**Pre-trigger samples**	State the number of pre-trigger samples or recording time.	Signals were recorded with 200 pre-trigger samples.
	**Raw signal constitution**	State whether the raw signal underwent some processing by the device.	At each measurement instance 10 signals were collected, and their average was computed to derive the raw signal.
	**Frequency spectrum of raw signal**	Report the frequency spectrum of the raw signal to document actual signal content and validate filtering decisions.	Figure with frequency spectrum.
Preprocessing	**Preprocessing steps**	List all preprocessing steps performed.	Offset compensation, crosstalk and pre-trigger removal and band pass filtering were performed.
	**Offset compensation method**	Specify the time window and whether mean or median was used.	Offset compensated using mean amplitude of the pre-trigger samples ($t_{1}^{off}$ = 0 µs, $t_{2}^{off}$= 100 µs).
	**Crosstalk removal window**	Report the time window used to remove crosstalk, and whether crosstalk was overlapping with the main signal.	Crosstalk occurred before the main signal. For crosstalk removal, the values within 0 µs to 125 µs were set to zero.
	**Pre-trigger removal window**	State the time window used for pre-trigger removal.	The pre-trigger samples (0 µs to 100 µs) were removed.
	**Bandpass filter specifications**	Report filter type as detailed as possible.	A zero-phase Butterworth bandpass filter (order N) was applied.
	**Frequency range for filtering**	Specify which frequency band(s) were used and justify the choice.	Two ranges applied: 10 kHz – 150 kHz, and 40 kHz – 150 kHz (to exclude 25 kHz mode).
	**Oversampling**	If implemented, report target sampling rate, and interpolation method.	Oversampling to 10 MHz was applied by zero-padding to improve resolution for small α increments.
Coda Wave Interferometry	**CWI technique**	Report CWI technique and equations used.	See Section 3 for details.
	**Signal window**	Report the time window(s) used for calculation.	Signal windows 0 µs – 250 µs, 0 µs – 1250 µs, and 0 µs – 5000 µs were investigated.
	**Reference adopted**	Report whether fixed, stepwise, or moving reference was used, and justify the choice.	Fixed reference was used for initial analysis; stepwise reference was additionally applied because $CC_{max}$ < 0.7.
	**Range of α**	Specify the range of used α for computation.	Stretching factors of α ∈ (−0.075,0.075) were considered to cover expected velocity changes up to ±6.0 %.
	**Resolution of α/Increments of α**	Report the resolution of α and/or the number of increments.	Analysis was performed with 10,001 steps yielding a resolution of 1.5 ⋅ 10^–5^.

### Method validation

The proposed procedure is validated by application to ultrasonic signals collected from one concrete specimen subjected to compression loading with the measurement specifications stated above. These signals were generated and analyzed in a previous study by Diewald et al. [[10], [22]]. From experimental time *t*_exp_ = 0 min compressive load was increased continuously until failure with a loading rate of 50 N/s, corresponding to a stress increase of 0.005 N/mm^2^s. Ultrasonic signals were collected at an interval of 10 s leading to an increase in compressive stress of 0.05 N/mm^2^ between two adjacent signals. With a predetermined compressive strength of 42.9 MPa this equals a 0.1 % stress increase in terms of compressive strength. Further experimental details and data interpretation is given by Diewald et al. [10] and Vu et al. [28].

**Fig. 6:**
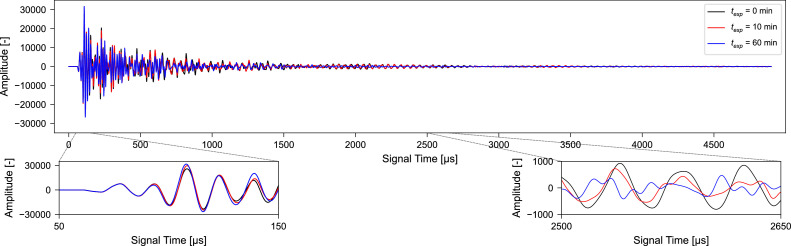
Preprocessed ultrasonic signals collected from one concrete specimen subjected to increasing compressive loads. Frequency filtering was performed for a frequency range of 10 kHz – 150 kHz. The compressive stress on the specimen was 0 N/mm^2^ at experimental time *t_exp_* = 0 min, 3 N/mm^2^ at *t_exp_* = 10 min, and 18 N/mm^2^ at *t_exp_*= 60 min.

The signals were preprocessed according to the workflow described herein (see also Fig. 3, Fig. 4). Frequency filtering is performed for both suggested frequency ranges 10 kHz – 150 kHz and 40 kHz – 150 kHz creating two sets of preprocessed signals which were subsequently analyzed by CWI methods. Fig. 6 displays three preprocessed ultrasonic signals collected during the experiment.

#### Influence of resolution of α

**Fig. 7:**
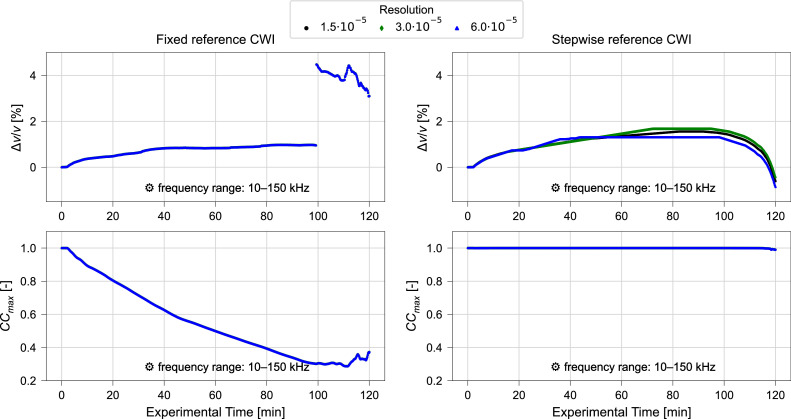
Relative velocity change Δv/v (upper) and maximized correlation coefficient $CC_{max}$ (lower) calculated using varying resolutions and fixed reference (left) or stepwise reference (right) CWI for ultrasonic signals collected during compression test. Frequency filtering performed for a frequency range of 10 kHz – 150 kHz. Results of CWI performed with α∈ (−0.075,0.075), for signal window 2500 µs. Data from Diewald et al. [[10], [22]].

**Fig. 8:**
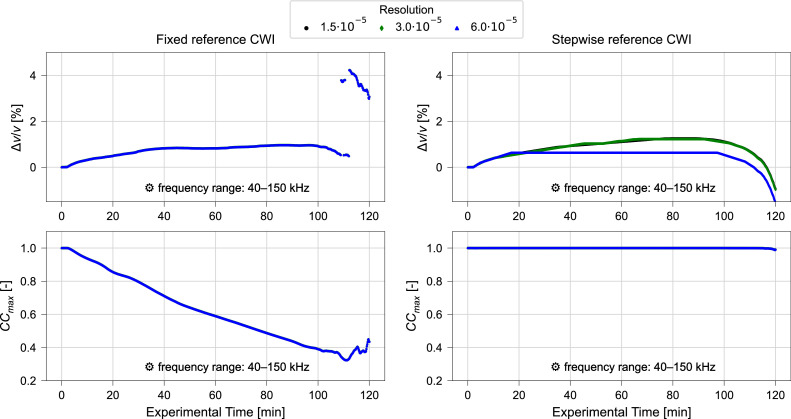
Relative velocity change Δv/v (upper) and maximized correlation coefficient (lower) calculated using different resolutions for *α* and fixed reference (left) or stepwise reference (right) CWI for ultrasonic signals collected during compression test. Frequency filtering performed for a frequency range of 40 kHz – 150 kHz. Results of CWI performed with α∈ (−0.075,0.075), for signal window 2500 µs. Data from Diewald et al. [[10], [22]].

The validity of the proposed resolution of α is tested by performing fixed and stepwise reference CWI with 2501, 5001, and 10,001 increments considered within the range of α with α∈(−0.075,0.075). The respective results for α = ∆*v*/*v* as obtained over the experiment duration are shown in Fig. 7, Fig. 8.

While no differences are observed for the fixed reference calculations, the results obtained by stepwise reference show differences depending on the applied resolution. For 6.0 ⋅ 10^–5^, a linear increase in ∆*v*/*v* between 20 min and 40 min and a prominent flat region between 40 min and 100 min is observed. This indicates that the true value is not covered by the set resolution. The shape of the curve changes when increasing from 6.0 ⋅ 10^–5^ to 3.0 ⋅ 10^–5^ aligning better with the smooth curved trend observed with fixed reference CWI. However, also for 3.0 ⋅ 10^–5^ a linear increase between 20 min and 70 min and a flat region between 70 min and 95 min is observed. The proposed resolution of 1.5 ⋅ 10^–5^ provides smoothest curve without prominent linear sections suggesting this resolution to sufficiently capture the true character of the signal change.

**Fig. 9:**
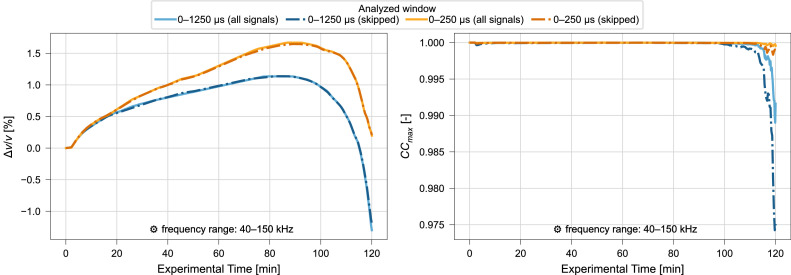
Relative velocity change Δv/v (left) and maximized correlation coefficient $CC_{max}$ (right) calculated using all signals of the dataset (solid) or skipping every second signal (dash dot). Results for signal windows of 0 µs – 250 µs and 0 µs – 1250 µs and stepwise reference CWI for ultrasonic signals collected during compression test. Frequency filtering performed for a frequency range of 40 kHz – 150 kHz. Results of CWI performed with range of α∈ (−0.075,0.075), for resolution 1.5⋅10^–5^. Data from Diewald et al. [[10], [22]].

The appropriateness of the proposed resolution of α with regard to error propagation in the stepwise reference approach is further explored by performing stepwise reference CWI once including all signals and once skipping every second signal of the dataset. The analysis was conducted with 10,001 increments considered within the range of α with α∈(−0.075,0.075) for a signal window of 0 µs – 250 µs and 0 µs – 1250 µs. In Fig. 9 the obtained Δv/v values are presented over the experiment duration. There is little difference between the two variants in Δv/v values and while apparent in the $CC_{max}$ values of the later part of the experimental period $CC_{max}$ stays at a high level above 0.97. These results suggest the stability and reliability of the results obtained with the proposed resolution of 1.5 ⋅ 10^–5^.

#### Influence of selected signal window

**Fig. 10:**
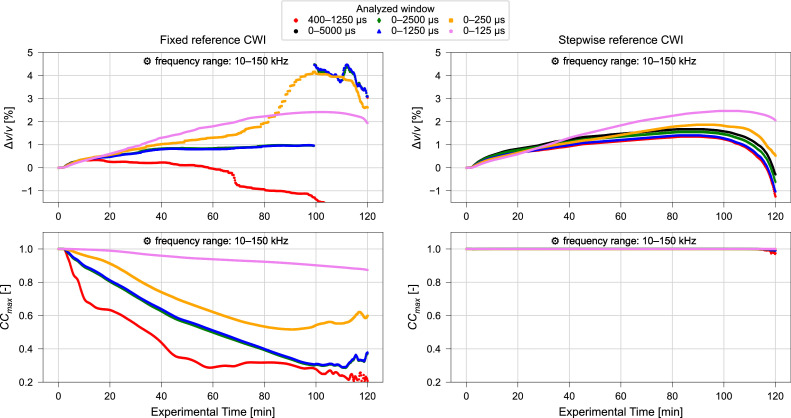
Relative velocity change Δv/v (upper) and maximized correlation coefficient $CC_{max}$ (lower) calculated using varying time windows and fixed reference (left) or stepwise reference (right) CWI for ultrasonic signals collected during compression test. Frequency filtering performed for a frequency range of 10 kHz – 150 kHz. Results of CWI performed with range of α∈ (−0.075,0.075) for resolution of 1.5⋅10^–5^. Data from Diewald et al. [[10], [22]].

**Fig. 11:**
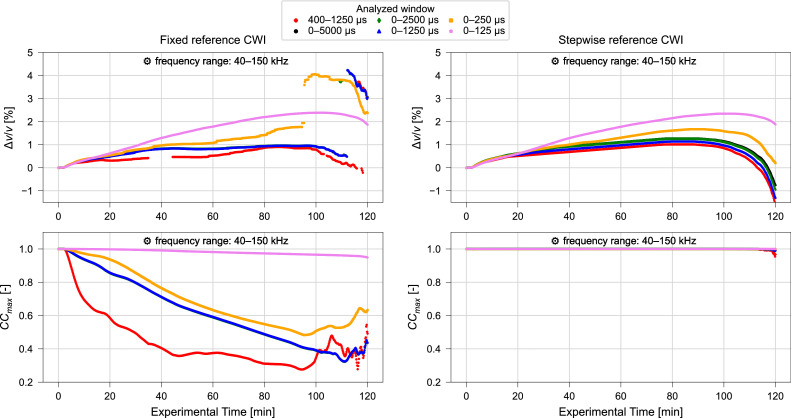
Relative velocity change Δv/v (upper) and maximized correlation coefficient $CC_{max}$ (lower) calculated using varying time windows and fixed reference (left) or stepwise reference (right) CWI for ultrasonic signals collected during compression test. Frequency filtering performed for a frequency range of 40 kHz – 150 kHz. Results of CWI performed with range of α∈ (−0.075,0.075), for resolution 1.5⋅10^–5^. Data from Diewald et al. [[10], [22]].

**Fig. 12:**
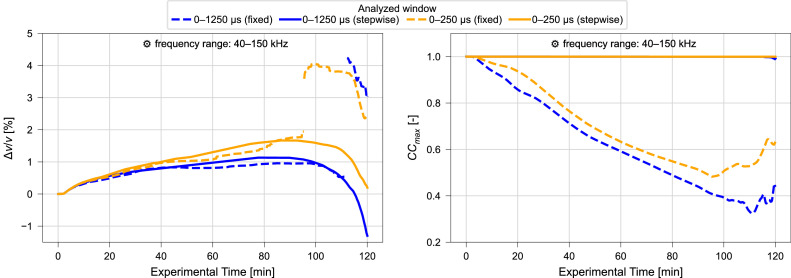
Relative velocity change Δv/v (left) and maximized correlation coefficient $CC_{max}$ (right) calculated using varying time windows and fixed reference (dashed) or stepwise reference (solid) CWI for ultrasonic signals collected during compression test. Frequency filtering performed for a frequency range of 40 kHz – 150 kHz. Results of CWI performed with range of α∈ (−0.075,0.075), for resolution 1.5⋅10^–5^. Data from Diewald et al. [[10], [22]].

The signal window is assessed by application of fixed reference and stepwise reference CWI for the signal windows of 0 µs – 125 µs, 0 µs – 250 µs, 0 µs – 1250 µs, 0 µs – 2500 µs, 0 µs – 5000 µs, and 400 µs – 1250 µs. The results obtained over the experiment duration are illustrated in Fig. 10, Fig. 11. For better comparison of fixed and stepwise reference the results obtained using the suggested windows of 0 µs – 250 µs and 0 µs – 1250 µs are additionally displayed together in Fig. 12.

Significant changes in the results are observed when extending the signal window from 0 µs – 125 µs to 0 µs – 250 µs. However, no prominent changes are observed when comparing the results acquired with 0–5000 µs to the results obtained with 0 µs – 2500 µs and 0 µs – 1250 µs. Therefore, it is suggested that consideration of longer signal windows than 0 µs – 1250 µs does not provide additional information.

Further, the results for the window of 0 µs – 1250 µs can be compared to the results calculated for the similar window 400 µs – 1250 µs which excludes the ballistic (direct) wave. When using the fixed reference CWI, the exclusion of the initial signal part leads to a significant decrease in the maximized correlation coefficients obtained. However, when using the stepwise reference approach, only marginal differences are observed. This suggests that, for the relatively small sample geometry shown, including the initial signal component is beneficial for stability and interpretability. For this sample size it is therefore recommended to use a window that starts at 0 µs. However, for larger or different geometries, effects may change, which is why a sensitivity analysis on the effect of the considered time window including both ballistic and coda and only the coda is required.

Whether 0 µs – 250 µs or 0 µs – 1250 µs should be used for interpretation should be evaluated by comparison with supplementary information, e.g., temperature or strain. However, with ultrasonic pulse wave velocities around 4300 m/s, the p-waves can travel the length of the specimen 2 – 3 times within the 0 µs – 250 µs period and around 13 times during the 0 µs – 1250 µs period, which can be assumed to be sufficient for the application of CWI. In these durations, the s-waves (velocity around 2500 m/s) will travel the length of the specimen 1 – 2 times and about 8 times respectively.

#### Influence of frequency filtering

Only minor variations in relative velocity change and maximized correlation coefficient are observed when adopting a frequency range of 10 kHz – 150 kHz (Fig. 7, Fig. 10) instead of the frequency range of 40 kHz – 150 kHz (Fig. 8, Fig. 11). Moreover, when comparing the results obtained for different signal windows illustrated in Fig. 10, Fig. 11 one might even argue that the variations induced by the change in frequency range are negligible. This demonstrates that both recommended frequency ranges deliver consistent results in this study.

#### Computational cost

**Fig. 13:**
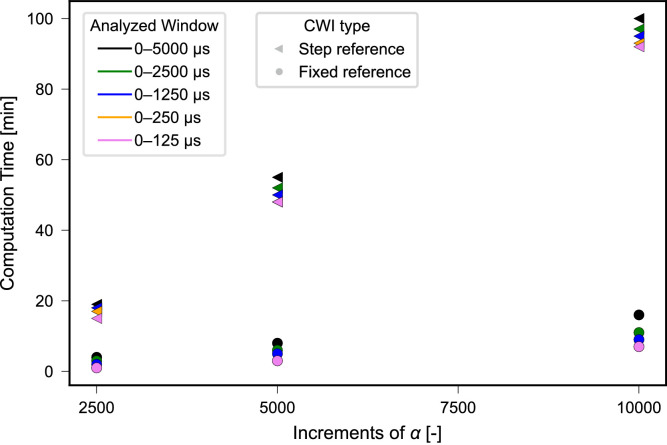
Computational costs of the CWI analysis in minutes required for computation when using fixed and stepwise CWI, varying signal windows and resolutions of α with α∈ (−0.075,0.075) and 2501, 5001, and 10,001 increments. The results of the CWI analysis are illustrated in Fig. 7, Fig. 10.

Actual computational costs depend not only on the above-mentioned analysis parameters set for calculation, but also on the software implementation and programming language, as well as the computer hardware used. Nevertheless, the computing times determined for the data shown in Fig. 7, Fig. 10 can be referred to in order to assess the influence of the analysis parameters. For the respective calculations, all steps presented in the Methods Section were implemented in Python and executed on an Intel Xenon Silver4114 processor. The computation times required for analysis are illustrated in Fig. 13.

The most prominent difference is observed when comparing fixed reference and stepwise reference CWI. While fixed reference CWI takes < 20 min for all considered variants, computational times of up to around 100 min are observed for stepwise reference CWI. This can be explained from the fact that for the stepwise reference, a completely new signal pair is compared each time, while for the fixed reference, one partner, the reference signal, remains the same for the full analysis. Therefore, the same set of pre-calculated stretched and compressed signals can be used. Thus, initial analyses and adjustment of parameters should be tested with the fixed reference first.

Moreover, it is observed that the computation time is strongly dependent on the increments of α. By reducing the number of increments from 10,001 to 5001, the computation time is cut by almost 50 %. This is a result of having to calculate a stretched signal for each α tested, which makes the computational time proportional to the increments of α. As discussed above, the resolution of α, i.e., the step size of the α values considered in the analysis, is of importance to obtain accurate data without artefacts (see also Fig. 7, Fig. 8). For the calculations presented in Fig. 7–Fig. 12 the range of α was fixed at α∈ (−0.075,0.075) as proposed in Section 3.2 based on expected relative velocity changes. The highest resolution is therefore obtained with 1.5∙10^–5^ for 10,001 increments of α. As this is considered to be more a lower boundary value and finer resolutions, i.e., increasing increments of α, would lead to even longer computation times, it is recommended to perform initial analysis with a resolution of 1.5∙10^–5^ at a range of α∈ (−0.075,0.075) and 10,001 increments. Further analysis can then be performed based on the initial results with a narrowed range of α and decreased number of increments keeping the resolution at 1.5∙10^–5^ or above while reducing computation time.

The choice of signal window is found to have a minor effect on the computation time. For stepwise reference, cutting the signal window from 0 µs – 5000 µs to 0 µs – 2500 µs reduces the computational costs by only up to 3 min. Therefore, initial analysis with fixed and stepwise reference and a signal window of 0 µs – 250 µs and 0 µs – 1250 µs provides robust initial results at reasonable costs.

## Limitations

The following limitations apply to the proposed method:

1. Specific experimental setup: The method is described for a specific specimen and hence the results must be revalidated for different geometry and test conditions.
2. Preprocessing parameters are dependent on the data acquisition system, so it is necessary to adapt to each preprocessing step accordingly.
3. Filtering parameter for bandpass filter might require tuning but must remain zero phased.
4. The analysis parameters must be re-evaluated in the context of the specific experiment to ensure that the CWI output is accurate.
5. Any results rely on the selection of the initial reference signal, which is not always trivial and should therefore be carefully considered.

## Ethics statements

None.

## CRediT author statement

**Eva Jägle**: Conceptualization, Methodology, Software, Validation, Formal analysis, Investigation, Data Curation, Writing - Original Draft, Writing - Review & Editing, Visualization, Project administration **Rujika Tuladhar**: Methodology, Software, Validation, Formal analysis, Investigation, Data Curation, Writing - Review & Editing, Visualization **Ernst Niederleithinger:** Methodology, Formal analysis, Writing - Review & Editing, Funding acquisition **Niklas Epple:** Methodology, Software, Validation, Writing - Review & Editing **Camila Andrea Sanchez Trujillo**: Methodology, Validation, Writing - Review & Editing **Christoph Gehlen:** Resources, Writing - Review & Editing, Funding acquisition **Jithender J. Timothy:** Conceptualization, Methodology, Software, Validation, Formal analysis, Investigation, Resources, Writing - Review & Editing, Supervision, Funding acquisition.

## Related research article

None.

## Declaration of competing interest

The authors declare that they have no known competing financial interests or personal relationships that could have appeared to influence the work reported in this paper.

The authors declare the following financial interests/personal relationships which may be considered as potential competing interests:

Eva Jägle, Ernst Niederleithinger, Niklas Epple, Camila Andrea Sanchez Trujillo, Christoph Gehlen and Jithender J. Timothy report financial support was provided by German Research Foundation (DFG).

Eva Jägle, Ernst Niederleithinger, Niklas Epple, Camila Andrea Sanchez Trujillo, Christoph Gehlen and Jithender J. Timothy report a relationship with German Research Foundation (DFG) that includes: funding grants.

Rujika Tuladhar, Christoph Gehlen and Jithender J. Timothy report financial support was provided by TUM Georg Nemetschek Institute Artificial Intelligence for the Built World (GNI).

Rujika Tuladhar, Christoph Gehlen and Jithender J. Timothy report a relationship with TUM Georg Nemetschek Institute Artificial Intelligence for the Built World (GNI) that includes: funding grants.

## Figures and Tables

**Table:** 

For	we derive	with	which is interpreted as
*α* > 0	∆*v/v* > 0	$\tilde{v}>v$	velocity increase
*α* < 0	∆*v/v* < 0	$\tilde{v}<v$	velocity decrease
see Eq. A.8	see Eq. A.9	see Eq. A.2	

## Data Availability

The data used are publicly available: Diewald, F. et al.: Data for: Impact of External Mechanical Loads on Coda Waves in Concrete [Data set]. Zenodo, (2025).https://doi.org/10.5281/zenodo.17880293

## Appendix

A.1 Derivation of Eq. (5)

The ultrasonic wave velocity within a medium can be derived by the distance between transmitter and receiver d and the time t which the wave needs to travel this distance:

(A.1) $$v=\frac{d}{t}$$

The velocity change in the medium between two experimental times $t_{exp,1}$ and $t_{exp,2}$ can be expressed through the relative velocity change Δv/v, i.e., the change in velocity between the initial ultrasonic wave velocity v and the velocity at the later time $\tilde{v}$ relative to the initial velocity:

(A.2) $$\Delta v/v=\frac{\tilde{v}-v}{v}$$

Given Eq. A.1 and Eq. A.2, the relative velocity change can be related to the travel time t of the wave with the initial wave velocity and the travel time $\tilde{t}$ with the changed velocity as follows:

With

$v=\frac{d}{t}$ and $\tilde{v}=\frac{d}{\tilde{t}}$, it follows that:

$$\Delta v/v=\frac{\tilde{v}-v}{v}=\frac{\frac{d}{\tilde{t}}-\frac{d}{t}}{\frac{d}{t}}=\left(\frac{d}{\tilde{t}}-\frac{d}{t}\right)\cdot \frac{t}{d}=\frac{d\cdot t}{\tilde{t}\cdot d}-\frac{d\cdot t}{t\cdot d}=\frac{t}{\tilde{t}}-1$$

Therefore, we obtain:

$$\Delta v/v=\frac{t}{\tilde{t}}-1$$

Which can be reformulated as:

(A.3) $$t=\tilde{t}\cdot \left(1+\Delta v/v\right)$$

The two ultrasonic signals $u(t_{sig})$ and $\tilde{u}\left(t_{sig}\right)$ collected at the receiver at the respective experimental times result from the superposition of multiple waves that have passed through the medium. For each $\tilde{t}\in \left[t_{sig,1},\ldots .,t_{sig,n}\right]$ we can obtain the time *t* for which the amplitude of the initial signal u(t) equals the amplitude of the signal after velocity change $\tilde{u}\left(\tilde{t}\right)$ using Eq. A.3:

$$u\left(t\right)=\tilde{u}\left(\tilde{t}\right)\;for\;t=\tilde{t}\cdot \left(1+\Delta v/v\right)$$

Assuming homogeneous change in the medium between times $t_{exp,1}$ and $t_{exp,2}$, e.g., due to temperature changes, all waves traveling the medium experience the same velocity change, independent of their wave path and distance traveled in the medium. This can be mathematically expressed as:

(A.4) $$\Delta v/v=const,\forall t_{sig}$$

Therefore with $t_{sig}=\tilde{t}$ this leads to:

(A.5) $$u\left(t_{sig}\cdot \left(1+\Delta v/v\right)\right)=\tilde{u}\left(t_{sig}\right)$$

In application, Δv/v is the unknown quantity that we aim to determine. To obtain Δv/v, we assess different values and compare, for which value the two signals are most similar.

We therefore introduce the stretching factor *α* and reformulate Eq. A.5 as follows:

(A.6) $$u\left(t_{sig}\cdot \left(1+\alpha \right)\right)=\tilde{u}\left(t_{sig}\right)$$

The similarity of two data sets *X* and *Y* with $x_{1},x_{2},\ldots ,x_{n}\in X$ and $y_{1},y_{2},\ldots ,y_{n}\in Y$, can be elaborated using Pearson’s correlation coefficient $CC_{X,Y}$, which can be calculated using the mean values $\bar{x}$ and $\bar{y}$:

$$CC_{X,Y}=\frac{\sum_{i=1}^{n}\left(x_{i}-\bar{x}\right)\cdot \left(y_{i}-\bar{y}\right)}{\sqrt{\sum_{i=1}^{n}{\left(x_{i}-\bar{x}\right)}^{2}}\cdot \sqrt{\sum_{i=1}^{n}{\left(y_{i}-\bar{y}\right)}^{2}}}$$

The correlation coefficient can take values between −1 and 1, i.e., $CC_{X,Y}\in \left[-1,1\right]$. Positive values thereby indicate positive correlation between the values of *X* and *Y*, i.e., increasing values of *X* are associated with increasing values of *Y* and a value of 1 indicating a perfect linear relationship between both data sets. Negative values analogously indicate negative correlation where increasing values of *X* are associated with decreasing values of *Y*.

When comparing preprocessed ultrasonic signals, the data points, i.e., the wave amplitudes, fluctuate around zero (see also offset compensation described in Section 2.1). Therefore, we can simplify with $\bar{x}=\bar{y}=0$ as follows:

$$CC_{X,Y}=\frac{\sum_{i=1}^{n}x_{i}\cdot y_{i}}{\sqrt{\sum_{i=1}^{n}{\left(x_{i}\right)}^{2}}\cdot \sqrt{\sum_{i=1}^{n}{\left(y_{i}\right)}^{2}}}$$

Which can be rewritten in terms of $u(t_{sig})$ and $\tilde{u}\left(t_{sig}\right)$:

(A.7) $$CC=\frac{\int_{t_{1}}^{t_{2}}u\left(t_{sig}\right)\cdot \tilde{u}\left(t_{sig}\right)dt_{sig}}{\sqrt{\int_{t_{1}}^{t_{2}}{\left(u\left(t_{sig}\right)\right)}^{2}dt_{sig}\cdot \int_{t_{1}}^{t_{2}}{\left(\tilde{u}\left(t_{sig}\right)\right)}^{2}dt_{sig}}}$$

We can now formulate the correlation coefficient stated in Eq. A.7 as a function of the stretching factor *α* using Eq. A.6:

(A.8) $$CC\left(\alpha \right)=\frac{\int_{t_{1}}^{t_{2}}u\left(t_{sig}\cdot \left(1+\alpha \right)\right)\cdot \tilde{u}\left(t_{sig}\right)dt_{sig}}{\sqrt{\int_{t_{1}}^{t_{2}}{\left(u\left(t_{sig}\cdot \left(1+\alpha \right)\right)\right)}^{2}dt_{sig}\cdot \int_{t_{1}}^{t_{2}}{\left(\tilde{u}\left(t_{sig}\right)\right)}^{2}dt_{sig}}}$$

The value of *α*, which maximizes the correlation according to Eq. A.8 is then the value, for which:

(A.9) α=Δv/v

This gives the following interpretation for results obtained by maximizing CC(α):

Given that this consideration only assumes homogeneous change in the medium (see Eq. A.4), any inhomogeneous changes, where not all waves traveling the medium experience the same velocity change, are not reflected in the derived Δv/v value and lead to a maximized correlation $CC_{max}<1$.

A.2 Derivation of Eq. (6)

For the stepwise reference approach, stretching yields the relative velocity change Δv/v between two adjacent signals. For derivation of the signal change with respect to the initial state the velocity variation needs to be cumulated. The cumulated stretching factor $\alpha_{c}$ equal to the cumulated relative velocity change Δv/v, for the n^th^ signal can be calculated by using the following relationships derived from Eq. (5) or Eq. A.8:

$$t_{sig,n-1}=t_{sig,0}\left(1+\alpha_{c,n-1}\right)$$

$$t_{sig,n}=t_{sig,0}\left(1+\alpha_{c,n}\right)$$

which can be combined with

$$t_{sig,n}=t_{sig,0}\left(1+\alpha_{c,n-1}\right)\left(1+\alpha_{n}\right)$$

to obtain:

$$t_{sig,0}\left(1+\alpha_{c,n}\right)=t_{sig,0}\left(1+\alpha_{c,n-1}\right)\left(1+\alpha_{n}\right)$$

Eliminating $t_{sig,0}$ yields the cumulative stretching factor:

(A.10) $$\alpha_{c,n}=\left\{ \begin{array}{cc} \alpha_{n}, & n=1\\ \left(\alpha_{c,n-1}+1\right)\left(\alpha_{n}+1\right)-1, & n>1 \end{array}\right.$$

## References

[1] Capineri L., Bulletti A. (2021). Ultrasonic guided-waves sensors and integrated structural health monitoring systems for impact detection and localization: a review. Sens. (Basel).

[2] Aranguren G., Monje P.M., Cokonaj V., Barrera E., Ruiz M. (2013). Ultrasonic wave-based structural health monitoring embedded instrument. Rev. Sci. Instrum..

[3] Komlos̆ K., Popovics S., Nürnbergerová T., Babál B., Popovics J.S. (1996). Ultrasonic pulse velocity test of concrete properties as specified in various standards. Cem. Concr. Compos..

[4] Kashif Ur Rehman S., Ibrahim Z., Memon S.A., Jameel M. (2016). Nondestructive test methods for concrete bridges: a review. Constr. Build. Mater..

[5] R. Snieder, The Theory of Coda wave Interferometry, Pure appl. Geophys. 163 (2006) 455–473. 10.1007/s00024-005-0026-6.

[6] Stähler S.C., Sens-Schönfelder C., Niederleithinger E. (2011). Monitoring stress changes in a concrete bridge with coda wave interferometry. J. Acoust. Soc. Am.

[7] Planès T., Larose E. (2013). A review of ultrasonic Coda Wave interferometry in concrete. Cem. Concr. Res..

[8] Niederleithinger E., Wang X., Herbrand M., Müller M. (2018). Processing ultrasonic data by Coda wave interferometry to monitor load tests of concrete beams. Sens. (Basel).

[9] E. Jägle, E. Niederleithinger, S. Grabke, G. Vu, N. Sträter, E. Saenger, N. Epple, H. Wiggenhauser, C. Sanchez-T., C. Felix, F. Diewald, M. Balcewicz, J. Timothy, M. Ahrens, P. Mark, K.-U. Bletzinger, G. Meschke, C. Gehlen, Interdisciplinary research on the application of new ultrasonic methods for improved structural health monitoring, Technical University of Munich, 2024.

[10] Diewald F., Epple N., Kraenkel T., Gehlen C., Niederleithinger E. (2022). Impact of external mechanical loads on coda waves in concrete. Mater. (Basel).

[11] Diewald F., Denolle M., Timothy J.J., Gehlen C. (2024). Impact of temperature and relative humidity variations on coda waves in concrete. Sci. Rep.

[12] Clauß F., Epple N., Ahrens M.A., Niederleithinger E., Mark P. (2020). Comparison of experimentally determined two-dimensional strain fields and mapped ultrasonic data processed by coda wave interferometry. Sens. (Basel).

[13] Clauß F., Epple N., Ahrens M.A., Niederleithinger E., Mark P. (2022). Correlation of load-bearing behavior of reinforced concrete members and velocity changes of coda waves. Mater. (Basel).

[14] Grabke S., Clauß F., Bletzinger K.-U., Ahrens M.A., Mark P., Wüchner R. (2021). Damage detection at a reinforced concrete specimen with coda wave interferometry. Mater. (Basel).

[15] Epple N., Sanchez-Trujillo C.A., Niederleithinger E., Biondini F., Frangopol D.M. (2023). Life-Cycle of Structures and Infrastructure Systems.

[16] Sträter N., Clauß F., Ahrens M.A., Mark P., Biondini F., Frangopol D.M. (2023). Life-Cycle of Structures and Infrastructure Systems.

[17] Epple N., Schumacher T., Murtuz A.K.M.G., Niederleithinger E., Dusicka P. (2025). Combined passive and active ultrasonic stress wave monitoring of a full-scale laboratory reinforced concrete bridge column subject to reverse-cyclic lateral loading. J Civ. Struct Health Monit.

[18] Niederleithinger E., Wolf J., Mielentz F., Wiggenhauser H., Pirskawetz S. (2015). Embedded ultrasonic transducers for active and passive concrete monitoring. Sens. (Basel).

[19] Fröjd P., Ulriksen P. (2017). Frequency selection for coda wave interferometry in concrete structures. Ultrasonics..

[20] Barroso D.F., Epple N., Niederleithinger E. (2021). A portable low-cost ultrasound measurement device for concrete monitoring. Inventions.

[21] Pilz M., Parolai S. (2012). Tapering of windowed time series. Dtsch. GeoForschungsZentrum GFZ.

[22] Diewald F., Epple N., Kränkel T., Gehlen C., Niederleithinger E. (2025).

[23] Virtanen P., Gommers R., Oliphant T.E., Haberland M., Reddy T., Cournapeau D., Burovski E., Peterson P., Weckesser W., Bright J., van der Walt S.J., Brett M., Wilson J., Millman K.J., Mayorov N., Nelson A.R.J., Jones E., Kern R., Larson E., Carey C.J., Polat İ., Feng Y., Moore E.W., VanderPlas J., Laxalde D., Perktold J., Cimrman R., Henriksen I., Quintero E.A., Harris C.R., Archibald A.M., Ribeiro A.H., Pedregosa F., van Mulbregt P. (2020). SciPy 1.0: fundamental algorithms for scientific computing in Python. Nat. Methods.

[24] Wang X., Chakraborty J., Niederleithinger E. (2021). Noise reduction for improvement of ultrasonic monitoring using coda wave interferometry on a real bridge. J Nondestruct Eval.

[25] Sens-Schönfelder C., Wegler U. (2006). Passive image interferometry and seasonal variations of seismic velocities at Merapi Volcano, Indonesia. Geophys. Res. Lett..

[26] DIN Deutsches Institut für Normung e. V. (2020). Quantities and units – Part 2: mathematical signs and symbols to be used in the natural sciences and technology: (ISO 80000-2:2019).

[27] Sun X., Yi S., Li X., Cui Y., Zhang M., Jiang T., Wang L., Kong Q. (2026). A novel ultrasonic-velocity-based concrete carbonation monitoring method based on step-wise stretching technique. Struct. Health Monit..

[28] Vu G., Timothy J.J., Saenger E.H., Gehlen C., Meschke G. (2025). A virtual lab for damage identification in concrete using coda wave interferometry. Struct. Infrastruct. Eng..

[29] Planès T., Larose E., Rossetto V., Margerin L. (2013).

[30] Zhang T., Sens-Schönfelder C., Epple N., Niederleithinger E. (2022). Imaging of small-scale heterogeneity and absorption using adjoint envelope tomography: results from laboratory experiments. JGR Solid Earth.

[31] Kanu C., Snieder R. (2015). Time-lapse imaging of a localized weak change with multiply scattered waves using numerical-based sensitivity kernel. JGR Solid Earth.

[32] Larose E., Hall S. (2009). Monitoring stress related velocity variation in concrete with a 2 × 10(-5) relative resolution using diffuse ultrasound. J. Acoust. Soc. Am.

[33] Wang X., Niederleithinger E., Lange M., Stolpe H. (2019).

